# Examining Approaches to Estimate the Prevalence of Catastrophic Costs Due to Tuberculosis from Small-Scale Studies in South Africa

**DOI:** 10.1007/s40273-020-00898-3

**Published:** 2020-04-01

**Authors:** Sedona Sweeney, Anna Vassall, Lorna Guinness, Mariana Siapka, Natsayi Chimbindi, Don Mudzengi, Gabriela B. Gomez

**Affiliations:** 1 Department of Global Health and Development, London School of Hygiene & Tropical Medicine, Room 327, 15–17 Tavistock Place, London, WC1H 9SH UK; 2 Africa Health Research Institute, Durban, South Africa; 3 The Aurum Institute, Johannesburg, South Africa; 4 Vaccine Epidemiology and Modelling, Sanofi Pasteur SA, Lyon, France

## Abstract

**Background and Objective:**

In context of the End TB goal of zero tuberculosis (TB)-affected households encountering catastrophic costs due to TB by 2020, the estimation of national prevalence of catastrophic costs due to TB is a priority to inform programme design. We explore approaches to estimate the national prevalence of catastrophic costs due to TB from existing datasets as an alternative to nationally representative surveys.

**Methods:**

We obtained, standardized and merged three patient-level datasets from existing studies on patient-incurred costs due to TB in South Africa. A deterministic cohort model was developed with the aim of estimating the national prevalence of catastrophic costs, using national data on the prevalence of TB and likelihood of loss to follow-up by income quintile and HIV status. Two approaches were tested to parameterize the model with existing cost data. First, a meta-analysis summarized study-level data by HIV status and income quintile. Second, a regression analysis of patient-level data also included employment status, education level and urbanicity. We summarized findings by type of cost and examined uncertainty around resulting estimates.

**Results:**

Overall, the median prevalence of catastrophic costs for the meta-analysis and regression approaches were 11% (interquartile range [IQR] 9–13%) and 6% (IQR 5–8%), respectively. Both approaches indicated that the main burden of catastrophic costs falls on the poorest households. An individual-level regression analysis produced lower uncertainty around estimates than a study-level meta-analysis.

**Conclusions:**

This paper presents a novel application of existing data to estimate the national prevalence of catastrophic costs due to TB. This type of model could be useful for researchers and policy makers looking to inform certain policy decisions; however, some uncertainties remain due to limitations in data availability. There is an urgent need for standardized reporting of cost data and improved guidance on methods to collect income data to improve these estimates going forward.

**Electronic supplementary material:**

The online version of this article (10.1007/s40273-020-00898-3) contains supplementary material, which is available to authorized users.

## Key Points for Decision Makers

**Table:** 

The presented cohort model approach to estimate the national prevalence of catastrophic costs due to tuberculosis (TB), adjusting for variability across studies to reflect national demographics and loss to follow-up along the patient pathway of care, allows for the costs of those in care to be captured more accurately.
This approach facilitates estimation of the prevalence of catastrophic costs due to TB and the uncertainty of these estimates, and can identify the comparative impact of TB-related costs on different sections of the population.
Depending on the policy application, this approach could serve as a feasible alternative to country-wide national surveys to estimate catastrophic costs due to TB, where there are sufficient existing data available.

## Introduction

Tuberculosis (TB) remains the leading cause of death from a single infectious agent worldwide, with 10 million people falling ill and 1.2 million people dying from TB in 2018 [1]. Often those who are most affected by TB are the most vulnerable in society, and affected households can face substantial costs associated with the disease [2]. Globally, costs associated with TB represent an average of 58% and 39% of individual and household income, respectively [3].

In recognition of the impact of the costs of illness on households, the World Health Organization (WHO) has highlighted reduction of catastrophic costs due to TB as one of three priority targets for 2020 [1]. Costs due to TB are defined as ‘catastrophic’ by the WHO Global TB Programme where they exceed 20% of a household’s annual pre-TB income [4]. The focus of this metric is on economic hardship associated with seeking TB care, including direct out-of-pocket medical costs (such as money paid for medicines, diagnostics, consultation fees or informal payments made to health workers), direct non-medical costs (transport and accommodation costs, costs of any special food or supplements taken because of illness) as well as indirect (opportunity) costs of time spent seeking care by people with TB and guardians or household members accompanying them [5–8].

To help track country progress against this goal and inform programme planning, the Global TB Programme has developed guidelines for the conduct of nationally representative cross-sectional surveys to estimate the prevalence of catastrophic costs [4]. However, these surveys require ample resources and time to complete and will not be feasible for all 130 WHO member states to carry out repeatedly, leaving countries searching for another source of estimates.

In many settings, data on patient costs of TB have been collected as part of trials or other smaller-scale projects; however, recent systematic reviews of patient-incurred costs due to TB observed large heterogeneity in the quality of reporting as well as the methods used to collect cost data and measure income loss [3, 10, 11]. Given this variation, it is currently unclear to what extent this existing data can be used to inform national estimates of catastrophic costs due to TB. We hypothesize that with the use of a cohort model these data could still be a useful resource for countries looking for decision-making support, in the absence of a national survey. We aim to investigate approaches to model the national prevalence of catastrophic costs due to TB using the case study of South Africa, which has one of the world’s highest TB incidence rates, with an estimated incidence of 520 per 100,000 people in 2018 [12].

## Methods

### Parameterizing the Cohort Model: Population Characteristics

**Fig. 1:**
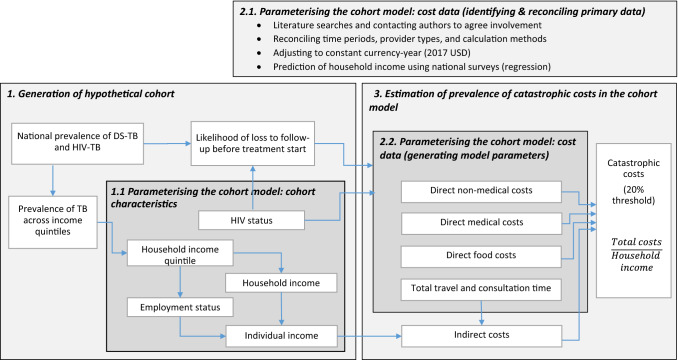
Analysis structure. *DS* drug-susceptible, *TB* tuberculosis

We created an individual-level deterministic cohort model that simulated progression through the TB care cascade in order to estimate the prevalence of catastrophic costs in South Africa (Fig. 1). The model contained a hypothetical cohort of 1000 South Africans with drug-susceptible (DS) TB, with population characteristics mirroring those of the national population of people with DS-TB. Individuals in the cohort were first distributed across national income quintiles 1–4 using data on the national income distribution and distribution of TB across income quintiles [13–15]. We then sampled employment status by income quintile, and household size reflecting national distributions of each [16]. Individual income was estimated by dividing household income by household size; individual income took a value of zero if unemployed or otherwise not income-earning. HIV sero-status was modelled for each individual in the cohort based on the national HIV prevalence among individuals with DS-TB [17]. We then estimated the likelihood of loss to follow-up before treatment start based on HIV status, following evidence from Naidoo et al. [17].

### Parameterizing the Cohort Model: Cost Data

#### Identifying and Reconciling Primary Data

We collated all research articles reporting any estimates of patient-incurred costs due to TB in South Africa from the Unit Cost Study Repository (UCSR) of the Global Health Cost Consortium [18]. Patient-incurred costs included any costs paid out-of-pocket by TB patients and their households, and any lost income or productivity due to TB. Eleven studies presenting patient cost data in South Africa were identified [19–29]. Of these, four were excluded due to outdated models of care and one was excluded as a duplicate of previously published data. Corresponding authors of seven eligible studies were invited to participate, and a protocol identifying variables to be included in the pooled dataset was provided. Collaborators from three of the seven eligible studies agreed to participate in the analysis. Due to data availability, the scope of this analysis was restricted to costs whilst on treatment for DS-TB; we did not consider costs for drug-resistant (DR) TB, nor did we consider costs during the diagnostic process [10]. All datasets had obtained ethical approval for their original study. Ethical approval for the pooled analysis was granted by the London School of Hygiene and Tropical Medicine (reference 14486).

We reconciled timeframes for cost data by identifying the treatment start date, interview date and recall period for each participant. Direct out-of-pocket costs incurred in each treatment phase (intensive and continuation phase) were categorized as direct medical costs (consultation fees, medicines, diagnostics), direct non-medical costs (transportation, accommodation) and food costs (food supplements, special foods). Cost estimates were distinguished by treatment phase (intensive and continuation phase) and by type of healthcare provider, including public healthcare (PHC) facility (study site), another PHC facility (non-study site), private general practitioner, pharmacy, hospital inpatient service, hospital outpatient service, and traditional healer.

All data in different studies were collected using adaptations of the Tool to Estimate Patient Costs [30], and thus definitions for out-of-pocket cost variables were homogeneous; however, the Researching Equity in ACcess to Healthcare (REACH) dataset did not contain information on direct non-medical costs or time spent accessing providers other than the main study clinic. As this was omitted entirely from data collection, we assumed these values to be missing at random and used imputation to complete these costs (imputation methods described in Sect. 2.2.2). In contrast, methods for collecting data on income and estimating indirect costs varied widely across datasets and were not reconcilable. To complete the datasets, we took a statistical approach to predict income quintile for households in the dataset. Assuming income distribution to be the same as the national distribution of income amongst people with TB, we used regression coefficients from an analysis run on the most recent (2015) South African National Income Dynamics Survey (NIDS) for variables including asset holdings, housing quality indicators and basic demographics to predict income. Full methods to predict household income quintiles are described in Electronic Supplementary Material (ESM) Appendix 2.

All costs are reported in 2017 US dollars. Data collected before 2017 were inflated using the US consumer price index [31]. Prior to generating model parameters using the standardized data, we conducted a descriptive analysis of sociodemographic and cost variables within and across datasets. Variables were summarized using the mean and standard deviation for each individual dataset and across the pooled dataset. We tested for significant differences in categorical variables using a chi-squared test, and tested for significant differences in continuous variables using a one-way analysis of variance (ANOVA) within and between studies.

#### Generating Model Parameters

We tested two approaches to estimate mean and standard error values for direct costs and hours lost due to treatment by household income quintile, HIV status and treatment phase: (1) meta-analysis of summary statistics from the standardized datasets; and (2) regression analysis of the pooled standardized dataset.

Our first approach was meta-analysis to calculate pooled estimates of available (study-level) mean values for the above-described cost categories for each treatment phase, by HIV status and household income quintile [32]. Given that patient demographics varied significantly across datasets, and assuming that patient costs vary according to demographics, we used a random effects meta-analysis approach, which does not assume that all studies investigate the same population [32]. Data on direct costs, travel time and consultation time were log-transformed for the meta-analysis as they were highly skewed, and results were exponentiated following meta-analysis.

Our second approach was to identify a regression model to predict the above-described cost categories for each treatment phase, by HIV status and household income quintile. Firstly, we imputed missing values in the pooled dataset. Where total consultation hours were missing, we used multivariate imputation with chained equations (MICE) to impute these values based on the number of visits by phase and provider type. Total travel hours and total direct non-medical costs were imputed based on number of visits and transport method, as well as demographic variables included in the regression analysis. All imputations used predictive mean matching (PMM), as a non-parametric alternative for imputing skewed data. Imputations generated 20 plausible datasets, which were then used for analysis. The number of missing observations by dataset are listed in ESM Table 3 [33].

Following imputation, we conducted a series of regression analyses to predict the cost of each cost category for each treatment phase. The regression analyses used a generalized linear model (GLM) approach assuming a gamma distribution and a log link to accommodate skewed data [34]. The specification of each regression was held constant across analyses and included independent variables identified following theory, as well as previous published evidence [35–40]. Independent variables were defined as urbanicity (1 = rural), education level (1 = educated to grade 8 and above), employment status (1 = employed), HIV status (1 = HIV positive) and household income quintile (quintiles 1–5). An interaction between employment status and income quintile was also included.

Following the regression analysis, marginal estimates for each of the above-described cost variables were obtained by HIV status, household income quintile and employment status, with urbanicity and education values held constant at the mean observed among people with TB in the NIDS dataset (urbanicity = 0.327; education above grade 8 = 0.683). To help us interpret the results of the regression analysis using the pooled dataset, we also tested the extent to which cost drivers varied across datasets. To do this, we conducted a regression on the unimputed data for each dataset separately. As there were insufficient observations to do this for the intensive phase, we conducted this test only on observations in the continuation phase.

### Estimation of Prevalence of Catastrophic Costs in the Cohort Model

We sampled patient-incurred direct costs and total time per individual in the cohort following a gamma distribution based on the mean and standard error values determined in the analyses described earlier. Individuals that were lost to follow-up before treatment initiation were assumed to encounter only costs of special food and supplements, while those initiated onto treatment were assumed to also encounter direct medical, direct non-medical and indirect costs.

Following prediction of total time spent accessing care, indirect costs were estimated using a human capital approach to value the opportunity cost of travel and consultation time whilst seeking care. This was calculated as the total hours lost multiplied by an approximation of individual income per hour, estimated assuming 220 working days per year and an 8-h working day. Indirect costs were assumed to be zero for those who were unemployed; however, we undertook a sensitivity analysis valuing costs for unemployed people using an equivalent individual salary for a similar person. Sensitivity analysis also tested inclusion of all treatment costs for those lost to follow-up. Due to a lack of data, we were not able to estimate indirect costs using an output approach (income loss due to illness), nor were we able to include costs incurred by other members of the household such as guardians or lay caregivers.

Catastrophic costs were defined as where the total patient-incurred cost during treatment was greater than 20% of annual household income [4]. For each model run, the prevalence of households encountering catastrophic costs was estimated per quintile and across the full cohort. The cohort model was simulated 10,000 times, to give 10,000 unique estimates of the national prevalence of catastrophic costs. Simulations were summarized using the median value and interquartile range (IQR) across all simulations.

## Results

### Demographic Characteristics, Direct Costs and Time Accessing Care in Published Studies

Three datasets were obtained and merged [27–29], resulting in a total of 1573 observations; 1219 were from the REACH study [27], 148 from the MERGE trial [29] and 171 from the XTEND (Xpert for TB: Evaluating a New Diagnostic) trial [28]. Table 1 shows the demographic data for each dataset, as well as the pooled dataset. Several demographic variables, including urbanicity, age, education and employment status, were significantly different across datasets. Although each study sample was randomly selected, the pooled datasets were not representative of the TB-affected population in South Africa as measured in the NIDS 2015 dataset.

**Table 1:** Summary demographic statistics from each of the datasets and the pooled data

Demographic statistic	REACH [27] (*n* = 1219)	MERGE [29] (*n* = 148)	XTEND [28] (*n* = 171)	Pooled dataset (*n* = 1573)	NIDS [16] (*n* = 244)^a^
Provinces	KwaZulu-Natal, Gauteng, Mpumalanga, Eastern Cape	Gauteng	Gauteng, Mpumalanga, Eastern Cape, Free State		
Income estimation methods	Self-reported household expenditure groups	Self-reported individual income	Self-reported individual income groups		
Total observations (*n*)					
Intensive phase	103	1	169	273	
Continuation phase	1049	146	170	1365	
Female [*n* (%)]	638 (52)	76 (51)	77 (45)	791 (51)	119 (39)
Urban [*n* (%)]	628 (52)	148 (100)	109 (63)	885 (58)	123 (35)
Mean age [years (SD)]	37 (12)	35 (10)	40 (13)	37 (12)	41.1 (13.0)
Black/African [*n* (%)]	1162 (95)	145 (98)	168 (98)	1475 (96)	212 (92)
Grade 8 and above^b^ [*n* (%)]	756 (62)	125 (84)	124 (72)	1005 (65)	138 (61)
Married/cohabitating [*n* (%)]	315 (26)	48 (32)	56 (33)	419 (27)	64 (23)
Employed at time of interview [*n* (%)]	195 (16)	75 (51)	64 (37)	334 (22)	82 (39)
Asset quintile distribution^b^ [*n* (%)]					
Q1	376 (31)	22 (15)	38 (22)	517 (29)	81 (33)
Q2	308 (25)	23 (16)	36 (21)	412 (23)	45 (18)
Q3	262 (21)	40 (27)	32 (19)	390 (22)	56 (23)
Q4	182 (15)	34 (23)	40 (23)	297 (17)	41 (17)
Q5	91 (7)	29 (20)	26 (15)	167 (9)	21 (9)
Coping strategies [*n* (%)]					
Coping	223 (18)	35 (24)	21 (12)	279 (18)	
Took loans	212 (17)	32 (22)	19 (11)	263 (17)	
Sold assets	26 (2)	7 (5)	5 (3)	38 (2)	

*n* number of observations, *NIDS* National Income Dynamics Survey, *Q* quintile, *SD* standard deviation

^a^Proportions weighted using survey weights to reflect the national average

^b^Education level of individual equivalent grade 8 or above

### Tuberculosis-Related Patient-Incurred Costs from Published Studies

Tables 2 and 3 show the meta-analysis results for direct medical costs, direct non-medical costs, food costs and hours lost (also see ESM Figures 1–5). The considerable uncertainty observed was a result of several factors, including the small numbers of observations, wide variation in visit frequency across datasets, and wide variation in costs within and between datasets. Availability of data also varied by dataset and treatment phase. ESM Table 1 lists the mean number of visits per month, direct costs per visit and time spent per visit by provider type and treatment phase for each of the datasets.

**Table 2:** Results of the meta-analysis: total direct medical and direct non-medical by household income quintile

Household income quintile	Total direct medical costs				Total direct non-medical costs											
	Intensive phase		Continuation phase		Intensive phase						Continuation phase					
	Other providers^a^		Other providers^a^		Other providers^a^		Study clinic		Food		Other providers^a^		Study clinic		Food	
	Mean	SE	Mean	SE	Mean	SE	Mean	SE	Mean	SE	Mean	SE	Mean	SE	Mean	SE
HIV negative																
Quintile 1	257.90	243.56	2.37	0.86	61.80	57.87	21.85	14.69	7.30	8.06	0.44	0.63	24.39	10.04	12.81	4.15
Quintile 2	17.64	187.03	12.96	1.09	11.08	4.38	6.87	1.36	16.38	15.32	2.11	0.60	8.14	9.30	47.38	26.57
Quintile 3	3.85	3.09	3.73	7.43	15.44	9.42	5.17	16.73	14.74	12.91	0.27	0.12	5.52	6.37	42.08	61.16
Quintile 4	3.97	55.34	4.06	4.99	7.82	4.65	5.63	3.86	3.00	53.08	0.14	0.08	11.21	10.71	30.61	83.59
Quintile 5	10.60	10.12	14.35	9.40	15.44	9.42	6.78	2.41	9.24	3.90	0.44	0.63	4.59	2.21	35.06	12.32
HIV positive																
Quintile 1	8.38	5.98	3.73	2.44	1.26	1.20	4.79	2.26	7.30	8.06	0.99	1.05	114.70	72.29	12.81	4.15
Quintile 2	2.88	5.19	4.66	3.45	3.91	0.86	5.55	2.32	16.38	15.32	0.71	3.48	12.07	6.21	47.38	26.57
Quintile 3	2.78	0.69	9.39	5.72	0.31	0.09	5.09	10.27	14.74	12.91	0.98	15.73	7.47	17.22	42.08	61.16
Quintile 4	9.18	51.63	7.19	7.07	1.83	0.72	4.74	33.66	3.00	53.08	0.59	1.97	9.38	10.32	30.61	83.59
Quintile 5	446.70	460.33	46.10	47.50	1.10	1.13	4.79	2.26	9.24	3.90	6.59	6.79	11.78	5.04	35.06	12.32

*SE* standard error

^a^Other providers: public healthcare facility (non-study site), private general practitioner, pharmacy, hospital (inpatient service), hospital (outpatient service) and traditional healer

**Table 3:** Results of the meta-analysis: total travel and consultation time by household income quintile

Household income quintile	Total travel and consultation time							
	Intensive phase				Continuation phase			
	Other providers^a^		Study clinic		Other providers^a^		Study clinic	
	Mean	SE	Mean	SE	Mean	SE	Mean	SE
HIV negative								
Quintile 1	23.25	22.44	4.96	2.90	0.57	0.49	2.42	47.72
Quintile 2	26.75	11.50	7.60	3.06	0.96	0.27	10.42	14.39
Quintile 3	0.08	0.04	14.76	2.24	0.19	0.13	30.13	4.57
Quintile 4	0.36	0.23	24.20	7.79	3.23	1.71	30.24	2.55
Quintile 5	2.05	11.63	10.86	2.46	0.57	0.49	4.00	1.03
HIV positive								
Quintile 1	14.29	5.17	5.55	4.31	1.19	2.04	4.61	1.35
Quintile 2	23.98	6.59	18.96	6.46	0.80	158.83	18.71	9.31
Quintile 3	8.08	2.42	2.54	23.08	0.98	0.80	42.12	4.63
Quintile 4	9.90	4.54	4.89	2.26	0.85	85.51	39.23	3.20
Quintile 5	30.17	31.08	5.55	4.31	8.58	8.85	25.73	4.81

*SE* standard error

^a^Other providers: public healthcare facility (non-study site), private general practitioner, pharmacy, hospital (inpatient service), hospital (outpatient service), and traditional healer

The variation in demographics observed across datasets provided a motivation for pursuing a regression analysis, which allowed inclusion of other explanatory variables in estimation of costs and time associated with accessing care. The results for the regression analysis are listed in Tables 4 and 5. Several independent variables were found to have a significant effect on cost. HIV status had a consistently positive effect. Being in a rural setting and having a higher education level both had a negative effect on direct costs and a positive effect on time spent accessing providers. Our tests of the regression model on the raw unimputed data separately for each dataset (ESM Tables 6–8) found some regression model coefficients were not consistent across datasets. There were no substantial differences observed in significant coefficients across datasets; where multiple datasets had significant coefficients for a given variable, coefficients were in the same direction and similar magnitudes.

**Table 4:** Results of the regression analysis: total direct costs and time lost; intensive phase

Variable	Intensive phase											
	Direct medical cost		Direct non-medical cost				Total travel and consultation time				Total cost for food or dietary supplements	
	Other providers^a^		Study clinic		Other providers^a^		Study clinic		Other providers^a^			
	Coeff	SE	Coeff	SE	Coeff	SE	Coeff	SE	Coeff	SE	Coeff	SE
HIV positive	− 0.484	0.611	− 0.444	0.381	0.0935	0.563	0.520*	0.206	3.457***	0.671	0.949***	0.278
Rural	− 0.871	0.510	− 0.700	0.426	− 0.683	0.486	0.151	0.234	− 0.248	0.748	− 1.416***	0.330
Grade ≥ 8	− 0.404	0.528	− 0.802*	0.388	− 0.307	0.583	0.130	0.208	1.565*	0.709	− 0.185	0.319
Unemployed; income quintile *(Reference Quintile 1)*												
Quintile 2	− 1.738	1.491	− 0.0569	1.062	− 1.958	1.437	0.410	0.599	− 3.983*	1.618	0.563	0.762
Quintile 3	− 2.710	1.452	0.285	1.104	− 3.015*	1.431	0.421	0.637	− 3.804*	1.624	1.065	0.857
Quintile 4	− 2.578	1.763	0.118	1.318	− 2.196	1.900	0.800	0.750	− 5.553**	1.960	1.854	0.969
Employed; income quintile *(Reference Quintile 1)*												
Quintile 2	0.657	1.548	− 0.0263	1.218	0.444	1.520	0.817	0.831	2.306	2.022	1.569	0.847
Quintile 3	− 1.219	1.599	0.460	1.200	− 2.147	1.617	0.499	0.663	− 3.616	1.930	1.734*	0.848
Quintile 4	− 0.0817	1.656	0.712	1.270	− 0.715	1.552	0.526	0.729	− 3.734*	1.827	2.013*	0.951
Quintile 5	3.155	2.570	1.843	2.107	− 2.287	2.462	0.00558	1.639	− 3.233	3.271	2.604	1.390
Constant	4.300	1.323	2.643**	0.996	2.871	1.271	− 0.351	0.567	0.256	1.481	2.135**	0.719
Observations	275		275		275		1539		1539		277	
*F*-statistic	4.03***		1.21		2.09*		1.08		6.95***		3.77***	
Degrees of freedom	10, 1.5e+11		10, 82,226		10, 2883		10, 3567		10, 12,995		10, 308,348	

*Coeff* coefficient, *SE* standard error

*p < 0.05; **p < 0.01; ***p < 0.001

^a^Other providers: public healthcare facility (non-study site), private general practitioner, pharmacy, hospital (inpatient service), hospital (outpatient service) and traditional healer

**Table 5:** Results of the regression analysis: total direct costs and time lost; continuation phase

Variable	Continuation phase											
	Direct medical cost		Direct non-medical cost				Total travel and consultation time				Total cost for food or dietary supplements	
	Other providers^a^		Study clinic		Other providers^a^		Study clinic		Other providers^a^			
	Coeff	SE	Coeff	SE	Coeff	SE	Coeff	SE	Coeff	SE	Coeff	SE
HIV positive	0.167	0.246	0.122	0.187	0.0787	0.338	0.203**	0.0742	0.723**	0.275	1.433***	0.205
Rural	− 1.033***	0.291	0.0700	0.210	− 0.748	0.387	1.190***	0.0893	0.361	0.290	− 0.923***	0.240
Grade ≥ 8	0.136	0.262	0.128	0.203	0.442	0.377	− 0.168*	0.0806	0.388	0.284	0.557*	0.220
Unemployed; income quintile *(Reference Quintile 1)*												
Quintile 2	1.750*	0.826	− 1.080	0.623	3.502**	1.078	− 0.0833	0.232	3.088***	0.899	0.265	0.647
Quintile 3	2.170*	0.868	− 0.983	0.664	3.918***	1.135	− 0.270	0.251	2.848**	0.920	0.364	0.692
Quintile 4	2.136*	0.954	− 0.578	0.739	4.152***	1.202	− 0.211	0.280	3.405**	1.040	1.198	0.763
Employed; income quintile *(Reference Quintile 1)*												
Quintile 2	1.661	0.986	− 0.522	0.751	2.095	1.227	0.169	0.293	2.518*	1.082	1.266	0.776
Quintile 3	2.422**	0.921	− 0.933	0.697	3.976***	1.183	0.0332	0.267	2.305*	0.995	1.174	0.719
Quintile 4	1.532	0.934	− 0.972	0.724	3.189**	1.220	− 0.290	0.279	2.400*	1.040	1.424	0.753
Quintile 5	3.046	1.637	− 2.635*	1.255	5.996**	1.933	− 1.702**	0.612	2.717	1.782	− 0.381	1.300
Constant	0.839	0.792	3.755***	0.602	− 1.518	1.051	2.445***	0.220	− 1.932*	0.846	2.509***	0.620
Observations	1339		1339		1339		1539		1539		1368	
*F*-statistic	2.00*		0.91		1.78		25.87***		3.27***		8.20***	
Degrees of freedom	10, 2.4e+08)		(10, 3.0e+06)		(10, 1057)		(10, 6428)		(10, 52,688)		(10, 2.7e+07)	

*Coeff* coefficient, *SE* standard error

*p < 0.05; **p < 0.01; ***p < 0.001

^a^Other providers: public healthcare facility (non-study site), private general practitioner, pharmacy, hospital (inpatient service), hospital (outpatient service) and traditional healer

### Catastrophic Costs in the Cohort Model

**Fig. 2:**
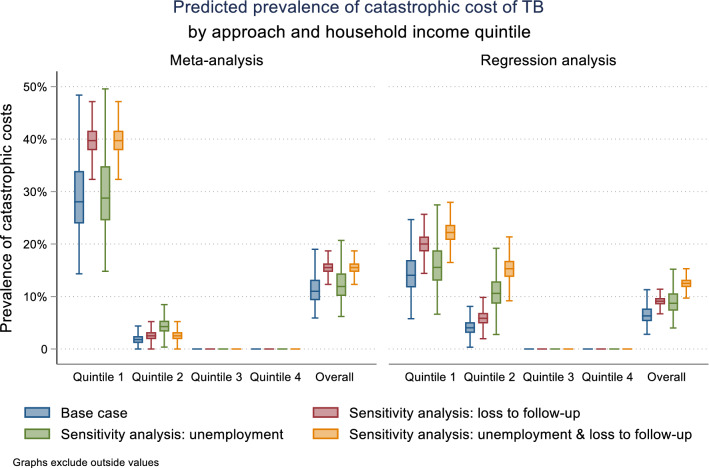
Prevalence of catastrophic cost of tuberculosis (TB) by approach and household income quintile (baseline results from 10,000 model runs)

Table 6 and Fig. 2 show the cohort model estimates of total costs and hours lost for both approaches, by household income quintile. Model estimates from both meta-analysis and regression approaches show the majority of the catastrophic cost burden falling on the first income quintile (meta-analysis: median 28%, IQR 24–34%; regression: median 14%, IQR 12–17%). Overall, 11% of people with TB nationally were predicted to encounter catastrophic costs using the meta-analysis approach (IQR 9–13%). Using inputs derived from the regression approach, the overall predicted prevalence of catastrophic costs was slightly reduced at 6% (IQR 5–8%).

**Table 6:** Cohort model results: total direct costs, time lost to accessing care, and prevalence of catastrophic costs by household income quintile and estimation approach

Quintile	Direct medical costs (study clinic and other providers^a^) (2017 $US)	Direct non-medical costs (2017 $US)		Travel and consultation time (study clinic and other providers^a^) (h)	Total indirect costs (2017 $US)	Annual household income (2017 $US)	Prevalence of catastrophic costs (%)
		Study clinic and other providers^a^	Special foods				
Meta-analysis approach							
Quintile 1	73.18 (61.95–88.74)	20.11 (19.79–20.42)	80.70 (68.53–97.13)	32.66 (27.73–40.04)	2.10 (1.66–2.61)	1315 (1289–1341)	28 (24–34)
Quintile 2	94.19 (80.32–114.98)	63.74 (62.51–64.98)	7.42 (6.28–9.07)	168.41 (142.86–206.19)	48.79 (39.94–59.64)	4156 (4120–4192)	2 (1–2)
Quintile 3	43.13 (35.40–52.34)	56.74 (53.60–59.89)	7.15 (6.04–8.73)	69.50 (58.70–84.55)	49.97 (40.31–60.97)	8385 (8299–8474)	0 (0–0)
Quintile 4	22.62 (18.18–29.43)	30.55 (26.24–35.04)	7.14 (5.40–9.34)	61.93 (52.58–75.25)	175.66 (137.10–225.11)	27,969 (26,176–30,050)	0 (0–0)
Overall	65.31 (55.48–79.70)	40.80 (39.73–41.87)	34.60 (29.39–41.68)	81.71 (69.54–100.15)	53.46 (43.76–65.58)	7858 (7503–8265)	11 (9–13)
Regression approach							
Quintile 1	48.26 (40.92–58.82)	9.12 (8.80–9.44)	24.23 (20.54–29.37)	61.47 (51.38–74.47)	3.48 (2.56–4.66)	1314 (1288–1340)	14 (12–17)
Quintile 2	29.03 (24.76–35.37)	26.64 (25.54–27.70)	27.16 (22.94–32.95)	212.10 (161.62–271.73)	134.04 (95.60–183.45)	4156 (4119–4192)	4 (3–5)
Quintile 3	28.70 (24.33–34.99)	38.36 (36.93–39.75)	17.97 (15.24–21.90)	16.83 (14.25–20.57)	12.13 (10.00–14.67)	8384 (8300–8471)	0 (0–0)
Quintile 4	32.99 (28.03–40.19)	64.23 (61.66–66.88)	21.64 (18.30–26.28)	16.22 (13.73–19.80)	37.99 (30.27–46.91)	27,993 (26,203–30,003)	0 (0–0)
Overall	36.67 (31.21–44.75)	28.58 (27.77–29.38)	23.36 (19.89–28.49)	88.00 (70.99–108.45)	47.57 (36.16–62.26)	7866 (7508–8259)	6 (5–8)

All data are given as median (IQR) US dollars

*IQR* interquartile range

^a^Other providers: public healthcare facility (non-study site), private general practitioner, pharmacy, hospital (inpatient service), hospital (outpatient service) and traditional healer

Including all costs for those lost to follow-up, and valuing time for those unemployed, increased the prevalence of catastrophic costs in quintiles 1 and 2 in both the meta-analysis and regression approaches. Our estimate of the overall prevalence of catastrophic costs using the meta-analysis approach were robust in sensitivity analyses; overall estimates for the regression approach increased slightly in sensitivity analysis.

## Discussion

We present estimates of the prevalence of catastrophic costs associated with TB, employing an individual-level cohort model using two approaches to parameterize cost estimates: a meta-analysis approach using study-level statistics and a regression approach using individual-level primary data. Overall, the median prevalence of catastrophic costs was estimated at 11% using a meta-analysis approach and 6% using a regression approach. Both approaches confirmed that catastrophic costs had a higher prevalence among the poorest quintile.

Our analysis had several limitations, mostly related to limitations in the available data. Due to limited data availability, the scope of the analysis was restricted to costs incurred by DS-TB patients during treatment. We were also unable to include costs before treatment initiation, costs incurred by other household members or indirect costs associated with lost income due to illness as there were no data on these costs. These restrictions on the analysis are likely to result in an under-estimation of the true prevalence of catastrophic costs. Furthermore, as all studies collected data at the health facility level, we were unable to include any cost estimates for people unable to access care. This may exclude the most vulnerable households impacted by TB, and thus likely results in an underestimate of the economic burden of TB. Finally, we do not include funeral costs for TB-related deaths, as there is limited evidence on these costs.

The meta-analysis approach produced higher estimates and wider uncertainty intervals than the regression analysis. This reflects the limitations of parameterization of a deterministic model using available cost and epidemiological data. We designed the model using the best available information on the TB epidemic in South Africa, using HIV status and income quintile to delineate TB prevalence and loss to follow-up rates. There was no available information on TB prevalence or loss to follow-up by urbanicity, unemployment or education level, and thus no way to include these effects in the meta-analysis approach.

In contrast, the better-parameterized regression approach allowed us to obtain cost estimates that had been corrected for the additional independent variables of urbanicity, unemployment and education. As these three variables were found to have significant effects on cost, it is therefore not surprising that the estimates obtained through the regression approach resulted in reduced uncertainty. This also explains the reduced estimated overall prevalence using the regression approach. Rural setting and higher education level both had a negative effect on direct costs, neither of which were accounted for in the meta-analysis.

Although the regression approach was better parameterized, our analysis indicates that better information is still needed on the household-incurred costs due to TB. Our findings on significant determinants of patient-incurred costs identified in the regression approach are largely supported by the existing literature [28, 36, 37]; however, these determinants were not consistently identified within the individual datasets and predictive power of the regression analyses were low. This may have been a consequence of small sample sizes and varying demographics across datasets; however, it could also reflect substantial differences in models of care in different settings. The extent to which TB and HIV services are integrated varies widely across South Africa [39]; this could lead to variation in the number of visits prescribed for those with both TB and HIV and thus a substantial difference in the degree to which positive HIV status is a driver of costs. Similarly, the extent to which directly observed therapy (DOT) is followed and its modality (facility-based or community-based) varies between settings. This is likely to have produced differences in cost drivers across datasets, for example increasing travel time for rural participants in settings where DOT is prevalent. To enable deterministic models such as the one presented here, further information is also needed on the healthcare pathway and drivers of care-seeking behaviour, including sub-national variations in models of care. This may also be evolving as the model of care for TB in South Africa continues to change.

Given these uncertainties, our analysis indicates that access to patient-level data is vital for researchers looking to extrapolate existing cost estimates to national settings, at least until the cost function can be better defined. The importance of improving data sharing is being increasingly recognized by publishers and funders [43, 44], not only to ensure transparency of research but also to maximize the benefit of data being collected. Primary data collection is often costly and time-consuming; the nationally representative WHO surveys are typically budgeted between $US27,000 and $US166,000 [4], while the costs of a secondary analysis are typically restricted to the time of the analyst (not including the costs of primary data collection in the original studies). Lengthy interviews about costs related to TB can also impose a substantial burden on patients and their household members, it is therefore important to make the most of data that are collected.

Of course, primary data collection also comes with some degree of uncertainty. Although primary cost data are increasingly being made available, for example through mechanisms such as the Global Health Cost Consortium’s UCSR [18], the wide variance in methods used to collect cost data remains a persistent limitation in the feasibility of pooling data. There are several areas where further data collection or better guidance on data collection methods would improve these estimates substantially. Firstly, methods for estimation and reporting of income data in patient-incurred cost surveys are currently inconsistent, with limited guidance on methods [41]. Going forward, better guidance on methods to estimate household and individual income is critical for any future attempts to pool data for drawing national estimates as well as more generally informing policy. Guidance on the appropriate measures of indirect costs in the numerator, and ability to pay in the denominator (e.g. household income vs. household expenditures), would also improve the theoretical validity of the metric [41].

Despite the above-discussed uncertainties, this type of model could be useful for researchers and policy makers. A cohort model such as the one presented in this paper can estimate the national prevalence of catastrophic costs due to TB and the uncertainty around these estimates, and can identify the comparative impact of TB-related costs on different sections of the population. It also has potential to inform certain policy decisions; for example, Verguet et al. [40] use a similar approach to illustrate the potential number of catastrophic costs averted from a range of TB interventions. The approach presented in this paper improves estimates by using a systematic approach to pool data from multiple studies, and allowing for adjustment of demographics and by treatment phase.

The usefulness of the type of analysis presented in this paper depends on the objectives of the analysis. This analysis may be sensitive enough to capture major movement towards the End TB goal of zero catastrophic costs due to TB; however, it is likely not sensitive enough to capture small changes from year to year—especially in settings where the cost function is still unknown or differs substantially in different settings. Ongoing primary data collection through national surveys is likely still necessary to facilitate annual reporting and programme management until the availability of cost and epidemiological data improves, and the cost function is better identified. However, while probably not providing quite as robust an estimate of catastrophic costs as a national survey, this type of analysis can complement, enrich and add depth to findings from the national surveys, especially for certain groups.

## Conclusions

This paper presents a novel use of existing data to estimate the prevalence of catastrophic costs due to TB [4]. We find that in the absence of nationally representative surveys, a deterministic model can provide an alternative for estimating catastrophic cost prevalence and the uncertainty around those estimates, with uncertainty slightly reduced using a regression approach as compared with a meta-analysis approach. A repeat of this analysis with additional primary data from South Africa added would test the validity of the main finding. Analyses testing the results of a cohort model against national estimates of catastrophic costs of other conditions would also help researchers to understand the validity of these models and the value of information added as compared with primary data collection through national surveys. Ultimately, to improve estimates from such cost-saving approaches, there is an urgent need for standardized methods to collect income data and standardized reporting of cost estimates.

## Electronic supplementary material

Below is the link to the electronic supplementary material.

Supplementary file1 (DOCX 520 kb)

## Data Availability

The dataset generated during the MERGE study is available in the LSHTM Data Compass repository at 10.17037/DATA.00001593. The dataset generated during the XTEND study is available in the LSHTM Data Compass repository at https://datacompass.lshtm.ac.uk/791/. The datasets generated during the REACH study are available from the corresponding author (NC) on reasonable request.
