# Supplementary material for: Examining Approaches to Estimate the Prevalence of Catastrophic Costs Due to Tuberculosis from Small-Scale Studies in South Africa
Source: Pharmacoeconomics. 2020 Apr 1;38(6):619–31. doi: 10.1007/s40273-020-00898-3 (PMC7307451; doi:10.1007/s40273-020-00898-3)
Supplement: Supplementary file 1 — Supplementary file1 (DOCX 520 kb) [file 40273_2020_898_MOESM1_ESM.docx]

## Examining approaches to estimate the prevalence of catastrophic costs due to tuberculosis from small-scale studies in South Africa

Running title: Estimation of TB-related catastrophic costs

Sedona Sweeney^a*^, Anna Vassall^a^, Lorna Guinness^a^, Mariana Siapka^a^, Natsayi Chimbindi^b^, Don Mudzengi^c^, Gabriela B Gomez^a, d^

^a^ London School of Hygiene & Tropical Medicine, Department of Global Health and Development

^b^ Africa Health Research Institute, South Africa

^c^ The Aurum Institute, South Africa

^d^ Sanofi Pasteur SA, Vaccine Epidemiology and Modelling, Lyon, France

Corresponding author: Sedona Sweeney, [sedona.sweeney@lshtm.ac.uk](mailto:sedona.sweeney@lshtm.ac.uk);

ORCID https://orcid.org/0000-0003-4233-9080

# Appendix 1: Supplementary Tables and Figures

Supplementary Table 1 Details of studies presenting household-incurred costs due to TB in South Africa

| Study | Year of  cost data collection | Provinces | Interventions | Sample size |
| --- | --- | --- | --- | --- |
| Wilkinson (1997) [19] | 1996 | KwaZulu-Natal | DS-TB treatment:  1) Hlabisa (current strategy); 2) Hlabisa (pre-1991 strategy); 3) Department of Health strategy; 4) SANTA strategy | 48 |
| Sinanovic (2003) [20] | 1998-9 | Western Cape | DS-TB treatment:  1) clinic-based care with community-based observation options; 2) clinic-based care only | 200 |
| Mandalakas (2013) [21] | No primary data | Not specified | IPT for young children in close contact with an infectious TB case |  |
| Sinanovic (2006) [22] | 2002-3 | North West, Free State, Western Cape | DS-TB treatment:  1) DOT in public-private workplace partnerships; 2) DOT in public-private non-government partnerships | 120 |
| Fairall (2010) [23] | 2003 | Free State | Educational outreach to primary care nurses | 1,999 |
| Van Rie (2013) [24] | 2010 | Johannesburg | Diagnosis of smear-negative TB with Xpert MTB/RIF | 199 |
| Du Toit (2015) [25] | 2013 | Western Cape | 1) MDR-TB diagnosis with LPA 2) MDR-TB diagnosis with XPERT | 153 |
| Ramma (2015) [26] | 2013 | Western Cape | Treatment of rifampicin-resistant and MDR-TB | 134 |
| Chimbindi (2015) [27] | 2009 | KwaZulu-Natal, Gauteng, Mpumalanga | Treatment of DS-TB | 1,219 |
| Foster (2015) [28] | 2012-13 | Gauteng, Mpumalanga, Eastern Cape, Free State | Diagnosis and treatment of DS-TB | 171 (cases);  35 (suspects) |
| Mudzengi (2016) [29] | 2013 | Gauteng | Treatment of DS-TB | 148 |

Supplementary Table 2 Cohort model inputs and distributions

|  | Mean | Std Err | Distribution | Source |
| --- | --- | --- | --- | --- |
| Number simulated iterations | 10000 |  | static |  |
| GINI index (2014) (G) | 0.63 |  | static | [42] |
| Annual per capita income | 10,130.10 |  | static | [15] |
| Household size | 4.65 | 3.27 | uniform | calculated from [16] |
| Risk of TB infection |  |  |  |  |
| DS-TB Overall |  |  |  |  |
| Annual burden | 507,533 | 101,742 | uniform | [17] |
| Accessed tests | 483,912 | 34,628 | uniform | [17] |
| Diagnosed | 417,277 | 12,639 | uniform | [17] |
| Notified and treated | 361,107 | 3,543 | uniform | [17] |
| Successfully treated | 274,441 | 55 | uniform | [17] |
| HIV-positive DS-TB |  |  |  | [17] |
| Annual burden | 314,491 | 76,913 | uniform | [17] |
| Accessed tests | 305,910 | 20,849 | uniform | [17] |
| Diagnosed | 257,316 | 7,793 | uniform | [17] |
| Notified and treated | 222,678 | 2,185 | uniform | [17] |
| Successfully treated | 164,804 | 1,674 | uniform | [17] |
| TB prevalence across quintiles |  |  |  |  |
| Quintile 1 | 0.37 |  | static | [13] |
| Quintile 2 | 0.28 |  | static | [13] |
| Quintile 3 | 0.18 |  | static | [13] |
| Quintile 4 | 0.17 |  | static | [13] |
| Quintile 5 | 0.00 |  | static | [13] |
| Frequency Employed |  |  |  |  |
| Quintile 1 | 0.27 | 0.02 | uniform | calculated from [16] |
| Quintile 2 | 0.38 | 0.01 | uniform | calculated from [16] |
| Quintile 3 | 0.47 | 0.01 | uniform | calculated from [16] |
| Quintile 4 | 0.57 | 0.01 | uniform | calculated from [16] |
| Quintile 5 | 0.64 | 0.02 | uniform | calculated from [16] |

##### Supplementary Table 3 Mean visits, costs, and time by dataset and treatment phase from the pooled primary data

|  | **Intensive phase** | | | | **Continuation phase** | | | |
| --- | --- | --- | --- | --- | --- | --- | --- | --- |
|  | **MERGE** | **REACH** | **XTEND** | **One-way ANOVA** | **MERGE** | **REACH** | **XTEND** | **One-way ANOVA** |
|  | **n = 1** | **n = 102** | **n = 172** | **(F statistic)** | **n = 146** | **n = 1021** | **n = 172** | **(F statistic)** |
| **Mean visits per month** | | | | | | | | |
| This clinic | 2.0 | 8.3 | 6.3 | 1.99 | 4.3 | 8.9 | 0.8 | 74.39*** |
| Pharmacy | 0.0 | 0.2 | 0.0 | 4.03* | 0.0 | 0.4 | 0.0 | 9.11*** |
| General Practitioner | 0.0 | 0.1 | 0.1 | 0.04 | 0.0 | 0.1 | 0.0 | 4.36* |
| Outpatient Hospital | 0.0 | 0.0 | 0.1 | 0.60 | 0.0 | 0.0 | 0.0 | 0.48 |
| Inpatient Hospital | 0.0 | 0.1 | 0.1 | 0.01 | 0.0 | 0.1 | 0.0 | 1.52 |
| Traditional Healer | 0.0 | 0.0 | 0.0 | 1.17 | 0.0 | 0.1 | 0.0 | 2.92 |
| **Mean direct medical cost per visit** | | | | | | | | |
| This clinic | $0.00 | $0.00 | $0.00 |  | $0.00 | $0.00 | $0.00 |  |
| Pharmacy |  | $2.42 | $54.13 | 2.50 | $0.22 | $1.84 | $7.13 | 5.02** |
| General Practitioner |  | $23.23 | $110.46 | 0.62 | $23.78 | $17.38 | $55.18 | 27.58*** |
| Outpatient Hospital |  | $7.28 | $40.05 | 0.11 | $4.12 | $2.87 | $4.63 | 0.45 |
| Inpatient Hospital |  | $0.00 | $104.72 | 0.15 | $18.69 | $1.14 | $13.46 | 4.00* |
| Traditional Healer |  |  | $90.37 |  | $439.05 | $20.58 | $109.76 | 139.02*** |
| **Mean direct non-medical cost per visit** | | | | | | | | |
| This clinic | $0.00 | $1.65 | $0.66 | 8.27*** | $1.00 | $2.06 | $1.14 | 1.39 |
| Pharmacy |  |  | $3.42 |  | $0.00 |  | $3.29 |  |
| General Practitioner |  |  | $6.88 |  | $26.56 |  | $4.28 | 1.91 |
| Outpatient Hospital |  |  | $12.66 |  | $9.88 |  | $5.39 | 0.76 |
| Inpatient Hospital |  |  | $24.39 |  | $17.57 |  | $5.43 | 0.60 |
| Traditional Healer |  |  | $14.63 |  | $21.95 |  | $0.00 | 0.06 |
| **Mean travel hours per visit** | | | | | | | | |
| This clinic | 1.0 | 0.7 | 0.6 | 0.06 | 1.2 | 0.6 | 0.9 | 55.95*** |
| Pharmacy |  |  | 0.5 |  | 1.9 |  | 0.2 | 3.33 |
| General Practitioner |  |  | 0.9 |  | 1.7 |  | 1.1 | 0.40 |
| Outpatient Hospital |  |  | 0.2 |  | 2.0 |  | 1.5 | 0.30 |
| Inpatient Hospital |  |  | 1.0 |  | 2.7 |  | 0.6 | 5.46* |
| Traditional Healer |  |  | 1.0 |  | 3.0 |  | 0.2 |  |
| **Mean consult hours per visit** | | | | | | | | |
| This clinic | 1.0 | 1.4 | 1.1 | 0.15 | 1.8 | 0.9 | 0.4 | 24.70*** |
| Pharmacy |  |  | 0.5 |  | 1.2 |  | 0.3 | 2.36 |
| General Practitioner |  |  | 1.1 |  | 1.5 |  | 0.9 | 1.97 |
| Outpatient Hospital |  |  | 2.7 |  | 5.3 |  | 2.6 | 7.85* |
| Inpatient Hospital |  |  | 126.3 |  | 104.0 |  | 26.4 | 3.80 |
| Traditional Healer |  |  | 0.6 |  | 9.0 |  | 13.2 |  |
| **Mean cost of ‘special foods’ or supplements** | | | | | | | | |
| Cost per phase | 27.44 | 4.21 | 15.60 | 7.80*** | 50.83 | 4.21 | 15.60 | 185.70*** |

##### Supplementary Table 4 Number of missing observations by dataset, phase, and provider type

|  | Intensive phase | | | Continuation phase | | |
| --- | --- | --- | --- | --- | --- | --- |
|  | MERGE | REACH | XTEND | MERGE | REACH | XTEND |
| Pharmacy |  |  |  |  |  |  |
| Direct medical cost | 0 | 0 | 1 | 1 | 0 | 1 |
| Direct non-medical cost | 0 | 103 | 0 | 1 | 1049 | 2 |
| Travel time (hours) | 0 | 103 | 0 | 2 | 1049 | 0 |
| Consult time (hours) | 0 | 103 | 0 | 2 | 1049 | 0 |
| General practitioner |  |  |  |  |  |  |
| Direct medical cost | 0 | 0 | 0 | 2 | 0 | 0 |
| Direct non-medical cost | 0 | 104 | 1 | 0 | 1047 | 1 |
| Travel time (hours) | 0 | 104 | 3 | 0 | 1047 | 1 |
| Consult time (hours) | 0 | 104 | 1 | 0 | 1047 | 1 |
| Hospital (inpatient) |  |  |  |  |  |  |
| Direct medical cost | 0 | 0 | 0 | 0 | 0 | 0 |
| Direct non-medical cost | 0 | 104 | 4 | 0 | 1050 | 1 |
| Travel time (hours) | 0 | 104 | 1 | 1 | 1050 | 1 |
| Consult time (hours) | 0 | 104 | 0 | 0 | 1050 | 0 |
| Hospital (outpatient) |  |  |  |  |  |  |
| Direct medical cost | 0 | 0 | 0 | 0 | 0 | 0 |
| Direct non-medical cost | 0 | 104 | 2 | 0 | 1050 | 2 |
| Travel time (hours) | 0 | 104 | 10 | 2 | 1050 | 3 |
| Consult time (hours) | 0 | 104 | 1 | 2 | 1050 | 3 |
| Traditional healer |  |  |  |  |  |  |
| Direct medical cost | 0 | 0 | 0 | 0 | 0 | 0 |
| Direct non-medical cost | 0 | 0 | 0 | 0 | 1046 | 0 |
| Travel time (hours) | 0 | 0 | 0 | 0 | 1046 | 0 |
| Consult time (hours) | 0 | 0 | 0 | 0 | 1046 | 0 |

##### Supplementary Figure 1 Meta-analysis results – total time (continuation phase)

##### Supplementary Figure 2 Meta-analysis results – total time (intensive phase)

##### Supplementary Figure 3 Meta-analysis results – direct medical costs

##### Supplementary Figure 4 Meta-analysis results – Direct non-medical costs (continuation phase)

##### Supplementary Figure 5 Meta-analysis results – Direct non-medical costs (intensive phase)

##### Supplementary Table 6 Regression results by dataset (Continuation phase only)

|  | Total Travel and Consultation Time | | | | | | |
| --- | --- | --- | --- | --- | --- | --- | --- |
|  | **Study Clinic** | | | | **Other Providers** | | |
|  | **MERGE** | **XTEND** | **REACH** | **Pooled dataset** | **MERGE** | **XTEND** | **Pooled dataset** |
| HIV positive | 0.28 (-0.14) | -0.08 (-0.18) | 0.199* (-0.08) | 0.203** (0.07) | 6.541*** (-1.67) | -12.14 (-13.35) | 0.723** (0.28) |
| Rural |  | -0.584** (-0.21) | 1.224*** (-0.09) | 1.190*** (0.09) |  | -3.90 (-2.87) | 0.36 (0.29) |
| Grade ≥ 8 | 0.07 (-0.18) | -0.07 (-0.19) | -0.15 (-0.08) | -0.168* (0.08) | 0.33 (-1.21) | 15.43 (-14.31) | 0.39 (0.28) |
| Unemployed; income quintile (ref: Q1) | | |  |  |  |  |  |
| Quintile 2 | 0.00 ((.) | 1.435* (-0.61) | -0.25 (-0.25) | -0.08 (0.23) | 0.00 ((.) | 5.22 (-13.42) | 3.088*** (0.90) |
| Quintile 3 | -0.23 (-0.21) | 1.467* (-0.64) | -0.45 (-0.26) | -0.27 (0.25) | 0.75 (-1.77) | 4.08 (-13.45) | 2.848** (0.92) |
| Quintile 4 | -0.639* (-0.29) | 1.26 (-0.69) | -0.24 (-0.30) | -0.21 (0.28) | -8.94E+16 ((.) | -2.38 (-13.03) | 3.405** (1.04) |
| Employed; income quintile (ref: Q1) | | |  |  |  |  |  |
| Quintile 2 | 0.28 (-0.43) | 1.14 (-0.63) | 0.14 (-0.32) | 0.17 (0.29) | -78.35 ((.) | 1.87 (-13.53) | 2.518* (1.08) |
| Quintile 3 | 0.00 (-0.22) | 1.22 (-0.63) | -0.28 (-0.28) | 0.03 (0.27) | -1.04 (-1.18) | 1.40 (-13.38) | 2.305* (1.00) |
| Quintile 4 | -0.30 (-0.21) | 1.793** (-0.69) | -0.619* (-0.31) | -0.29 (0.28) | 0.00 (-1.21) | -9.98 ((.) | 2.400* (1.04) |
| Quintile 5 |  | -5.07E+15 ((.) | -2.301*** (-0.54) | -1.702** (0.61) |  | 6.54 (-13.89) | 2.72 (1.78) |
| Constant | 3.644*** (-0.26) | -0.67 (-0.58) | 2.771*** (-0.23) | 2.445*** (0.22) | -4.123* (-1.94) | -3.79 (-13.48) | -1.932* (0.85) |
| Observations | 145 | 162 | 968 | 1539 | 146 | 172 | 1539 |

Standard errors in parentheses; * p<0.05, ** p<0.01, *** p<0.001

##### Supplementary Table 7 Regression results by dataset (Continuation phase only; continued)

|  | Total Direct Non-Medical Costs | | | | | | |
| --- | --- | --- | --- | --- | --- | --- | --- |
|  | **Study Clinic** | | | | **Other Providers** | | |
|  | **MERGE** | **XTEND** | **REACH** | **Pooled dataset** | **MERGE** | **XTEND** | **Pooled dataset** |
| HIV positive | 0.985* (-0.49) | 0.13 (-0.44) | 0.41 (-0.21) | 0.12 (0.19) | 22.52 ((.) | -0.94 (-1.87) | 0.08 (0.34) |
| Rural |  | -2.099*** (-0.54) | 0.14 (-0.21) | 0.07 (0.21) |  | -2.31 (-1.27) | -0.75 (0.39) |
| Grade ≥ 8 | -0.02 (-0.67) | -0.20 (-0.50) | 0.19 (-0.20) | 0.13 (0.20) | -8.29 (-49.22) | 1.34 (-2.22) | 0.44 (0.38) |
| Unemployed; income quintile (ref: Q1) | |  |  |  |  |  |  |
| Quintile 2 | 0.00 ((.) | -0.41 (-1.29) | -1.06 (-0.59) | -1.08 (0.62) | 0.00 ((.) | 2.69 (-2.17) | 3.502** (1.08) |
| Quintile 3 | -0.60 (-0.75) | -0.70 (-1.44) | -0.87 (-0.63) | -0.98 (0.66) | 6.36 (-49.23) | -21.18 ((.) | 3.918*** (1.14) |
| Quintile 4 | 5.70E-01 (-1.02) | 0.51 (-1.53) | -0.37 (-0.72) | -0.58 (0.74) | -1.52E+16 ((.) | 0.10 (-3.11) | 4.152*** (1.20) |
| Employed; income quintile (ref: Q1) | |  |  |  |  |  |  |
| Quintile 2 | 1.26 (-1.39) | -1.71 (-1.36) | -0.01 (-0.77) | -0.52 (0.75) | -9.64E+15 ((.) | 1.38 (-2.72) | 2.10 (1.23) |
| Quintile 3 | 0.81 (-0.73) | -1.28 (-1.39) | -0.83 (-0.69) | -0.93 (0.70) | 9.11 (-49.24) | 1.29 (-2.23) | 3.976*** (1.18) |
| Quintile 4 | 0.53 (-0.73) | 0.19 (-1.52) | -0.56 (-0.75) | -0.97 (0.72) | 7.26 (-49.24) | 0.36 ((.) | 3.189** (1.22) |
| Quintile 5 |  | -27.43 ((.) | -2.467* (-1.24) | -2.635* (1.26) |  | 5.63 (-3.37) | 5.996** (1.93) |
| Constant | 1.537* (-0.77) | 2.454* (-1.25) | 3.663*** (-0.57) | 3.755*** (0.60) | -20.65*** (-1.26) | -1.98 (-2.71) | -1.52 (1.05) |
| Observations | 146 | 142 | 1020 | 1339 | 146 | 172 | 1339 |

Standard errors in parentheses; * p<0.05, ** p<0.01, *** p<0.001

##### Supplementary Table 8 Regression results by dataset (Continuation phase only; continued)

|  | Total Direct Medical Costs | | | | Total cost for food or dietary supplements | | | |
| --- | --- | --- | --- | --- | --- | --- | --- | --- |
|  | **Other Providers** | | | |  |  |  |  |
|  | **MERGE** | **XTEND** | **REACH** | **Pooled dataset** | **MERGE** | **XTEND** | **REACH** | **Pooled dataset** |
| HIV positive | 18.29 (-3133.20) | -13.62 (-3276.40) | 0.42 (-0.28) | 0.17 (0.25) | 0.639* (-0.31) | 0.780** (-0.26) | -0.19 (-0.32) | 1.433*** (0.21) |
| Rural |  | -3.81 (-3.49) | -0.916** (-0.31) | -1.033*** (0.29) |  | -0.939** (-0.30) | -2.972*** (-0.35) | -0.923*** (0.24) |
| Grade ≥ 8 | -18.59 (-3133.20) | 16.58 (-3276.40) | 0.23 (-0.27) | 0.14 (0.26) | 0.07 (-0.41) | -0.01 (-0.30) | 0.842** (-0.33) | 0.557* (0.22) |
| Unemployed; income quintile (ref: Q1) | | |  |  |  |  |  |  |
| Quintile 2 | 0.00 ((.) | 6.60 (-3276.40) | 1.62 (-0.83) | 1.750* (0.83) | 0.00 ((.) | 0.55 (-0.87) | 0.80 (-0.84) | 0.27 (0.65) |
| Quintile 3 | -16.12 (-3133.20) | 7.11 (-3276.40) | 2.035* (-0.88) | 2.170* (0.87) | 0.01 (-0.47) | 0.75 (-0.96) | 0.85 (-0.90) | 0.36 (0.69) |
| Quintile 4 | -8.75E+15 ((.) | 3.87 (-3276.40) | 2.146* (-0.99) | 2.136* (0.95) | 0.77 (-0.63) | 1.43 (-0.98) | 0.48 (-1.06) | 1.20 (0.76) |
| Employed; income quintile (ref: Q1) | |  |  |  |  |  |  |  |
| Quintile 2 | -1.23E+16 ((.) | 6.94 (-3276.40) | 1.89 (-1.07) | 1.66 (0.99) | -0.06 (-0.88) | 0.77 (-0.90) | 0.92 (-1.13) | 1.27 (0.78) |
| Quintile 3 | 5.644*** (-1.34) | 6.51 (-3276.40) | 2.158* (-0.94) | 2.422** (0.92) | 0.28 (-0.47) | 1.17 (-0.91) | 1.50 (-0.98) | 1.17 (0.72) |
| Quintile 4 | 3.787** (-1.26) | -7.40 ((.) | 1.89 (-1.01) | 1.53 (0.93) | 0.31 (-0.47) | 1.41 (-0.99) | -0.66 (-1.06) | 1.42 (0.75) |
| Quintile 5 |  | 9.89 (-3276.40) | 2.48 (-1.74) | 3.05 (1.64) |  | 1.25 (-1.37) | -6.19E+15 ((.) | -0.38 (1.30) |
| Constant | -2.34 ((.) | -5.19 (-3276.40) | 0.87 (-0.80) | 0.84 (0.79) | 4.509*** (-0.53) | 3.620*** (-0.81) | 2.252** (-0.80) | 2.509*** (0.62) |
| Observations | 146 | 172 | 1050 | 1339 | 140 | 170 | 1050 | 1368 |

Standard errors in parentheses; * p<0.05, ** p<0.01, *** p<0.001

# Appendix 2: Methods for estimating income

This supplementary appendix describes in further detail methods for the regression used to predict income for the analysis presented in Chapter 9.

## Constructing the Asset Index

We first constructed an asset index using information on housing quality and ownership of durable assets [1]. The asset index was designed to reflect the relative socio-economic standing of households within South Africa as a whole, rather than the relative SES of households within the pooled dataset alone. We therefore used the South African National Income Dynamics Survey (NIDS) to draw weights for an asset index [2].

Vyas and Kumaranayake [3] recommend a principal components analysis (PCA) approach to estimate a wealth index, however, PCA was designed for use with continuous, normally-distributed variables and therefore its application to the categorical variables in a wealth index is considered by some to be inappropriate [4,5]. MCA is analogous to PCA but is designed for use with discrete data and was more appropriate to the type of asset data available in the dataset.

Inclusion of variables for the MCA model was tested before model finalization. The final model for the MCA included indicator variables for dwelling type, source of water, toilet type, main wall materials, and ownership of a number of durable assets including: a DVD player, a car, a radio, a television, a refrigerator, a cell phone, and a bicycle. Exploration with the MCA model indicated that inclusion of indicators of ownership of livestock and donkeys reduced the quality of the model rather than improved it; these were therefore left out of the final model. The MCA was conducted separately for rural and urban households, as asset ownership and inequality tend to be different in rural and urban areas [6].

The first dimension from the MCA explained 62.5% of variation in the dataset for rural households, and 73.4% of variation for urban households. Dimension weights were predicted using the Stata ‘predict’ command; dimension weights are listed in Table 1. Weights were largely positive for ownership of durable goods and indicators of high-quality housing (e.g. flush to sewage toilet, piped water inside dwelling), and negative for indicators of poor housing (e.g. no access to piped water, bucket toilet). Households in the NIDS dataset were classified into five socio-economic groups through splitting the dimension weight into five quintiles.

Coding for asset variables from the pooled dataset was then mapped to coding for the same questions from the NIDS, and weights from the MCA were applied to asset data in the pooled dataset. Using MCA weights, the position of households from the pooled dataset in the country-level SES quintiles were interpolated to reflect nationally-representative socio-economic quintile. The total number of households per quintile for each dataset is detailed in Table 9-1 in the main paper.

## Regression to predict income

We then used data from the NIDS dataset to predict coefficients for a number of demographic factors on household income and individual income.

Both household and individual income data were heavily right-skewed. In planning the regression we tested two regression approaches which have been recommended as appropriate for non-normally distributed data: a generalized linear model (GLM) with a gamma distribution and log link, and a quantile regression model [7].

Both regression models for household income were fit on covariates that are commonly included as determinants of income: urbanicity (1 = rural), gender (1 = female), education level (1 = educated to grade 8 and above), marital status (1 = married or cohabitating), employment status (1 = employed); asset quintile (quintiles 1-5, as described above), age group (1 = age 15-29; 2 = age 30-45; 3 = age > 45) and province. Following evidence that the burden of TB falls overwhelmingly on those with lower socioeconomic status [8,9], TB status (1 = current TB) was also included as a covariate in both regression models and the quantile regression model was fit on the log of household income at the 25^th^ quantile. Both regression models incorporated survey weights from the NIDS study calibrated to the corresponding population totals as given in the mid-year population estimates released in 2015 [10].

Robust standard errors were estimated in the quantile regression models to account for skewed data. Normality of residuals for both quantile regression and GLM models were tested using the Shapiro-Wilk normality test. The goodness of fit for a GLM is generally tested using the Akaike information criterion (AIC) and no R^2^ is reported for a GLM; direct comparison of the predictive power between the two models is therefore difficult. We report the pseudo R^2^ for the quantile regression model and AIC for the GLM.

Regression coefficients for both regression approaches (quintile and GLM) to estimate household income are listed in Supplementary Table 9-5. Coefficients for most covariates were significant, and there was little difference in coefficients across the two approaches. Tests after the quantile regression indicate that coefficients varied significantly across quantiles, suggesting that the quantile regression approach was more appropriate than the GLM approach. Supplementary Figure 9-7 shows the predicted coefficients for each covariate across quintiles. However, the predictive power for the quantile regression approach as indicated by the Pseudo R2 was relatively low (0.18), and the Shapiro-Wilk test indicates that residuals for both approaches deviate significantly from a normal distribution.

Coefficients from both regression analyses were used to predict the household income for patients in the pooled dataset, and correlation of predicted income and self-reported income variables were tested. Each dataset contained different self-reported income variables; correlation coefficients for predicted household income and income data collected in each dataset is listed in Supplementary Table 6. All correlation coefficients are relatively low; this is partly due to poor predictive power of the model, but also because most self-reported income variables were individual, whilst both regression approaches predicted household income. Most correlation coefficients were significant. There was relatively little difference in the size or significance of correlation coefficients between the quantile regression approach and the GLM approach.

The quantile regression approach was chosen as the best model, and income predictions using this model were used to classify households in the pooled analysis into nationally representative income quintiles.

## Predicted Household Income Quintiles

Coefficients for the regression to estimate household income are listed in Supplementary Table 5. Coefficients for most covariates were significant, and tests after the quantile regression indicate that coefficients varied significantly across quantiles. However, the predictive power for the quantile regression approach as indicated by the Pseudo R2 was relatively low (0.18), and the Shapiro-Wilk test indicates that residuals for the regression deviate significantly from a normal distribution.

Predicted income values were adjusted using a Duan smear factor [11], and households assigned to SES quintiles based on the adjusted predicted income using upper-income thresholds from Statistics South Africa. Only two per cent of observations from the pooled dataset fell into the first quintile, while most predictions fell into the second and third income quintile (46% and 38% respectively). In comparison, it has been estimated nationally that 37% of those with TB fall into the first quintile [8].

##### Supplementary Table 9 MCA results

|  | **Frequency by Dataset** | | | | **Urban** | | **Rural** | |
| --- | --- | --- | --- | --- | --- | --- | --- | --- |
|  | **AHRI** | **MERGE** | **XTEND** | **NIDS** | **Dimension 1 Coordinates** | **Contribution** | **Dimension 1 Coordinates** | **Contribution** |
| **Stove** |  |  |  |  |  |  |  |  |
| owns a Stove | 36% | 91% | 82% | 16% | 0.72 | 0.01 | 1.18 | 0.02 |
| does not own a Stove | 64% | 9% | 18% | 84% | -0.12 | 0.00 | -0.20 | 0.00 |
| **DVD player** |  |  |  |  |  |  |  |  |
| owns a DVD player | 45% | 74% | 63% | 37% | 0.92 | 0.03 | 1.54 | 0.05 |
| does not own a DVD player | 55% | 26% | 37% | 63% | -0.62 | 0.02 | -0.57 | 0.02 |
| **Motor car** |  |  |  |  |  |  |  |  |
| owns a Motor car | 12% | 19% | 19% | 19% | 1.64 | 0.05 | 2.36 | 0.06 |
| does not own a Motor car | 88% | 81% | 81% | 81% | -0.44 | 0.01 | -0.32 | 0.01 |
| **Radio** |  |  |  |  |  |  |  |  |
| owns a Radio | 75% | 77% | 80% | 63% | 0.49 | 0.01 | 0.53 | 0.02 |
| does not own a Radio | 25% | 23% | 20% | 37% | -0.77 | 0.02 | -0.86 | 0.02 |
| **Television** |  |  |  |  |  |  |  |  |
| owns a Television | 69% | 86% | 84% | 81% | 0.49 | 0.02 | 0.84 | 0.04 |
| does not own a Television | 31% | 14% | 16% | 19% | -2.37 | 0.08 | -2.14 | 0.11 |
| **Refrigerator** |  |  |  |  |  |  |  |  |
| owns a Refrigerator | 65% | 69% | 69% | 77% | 0.64 | 0.03 | 0.93 | 0.05 |
| does not own a Refrigerator | 35% | 31% | 31% | 23% | -2.26 | 0.09 | -1.91 | 0.10 |
| **Cell phone** |  |  |  |  |  |  |  |  |
| owns a cell phone | 83% | 99% | 96% | 90% | 0.19 | 0.00 | 0.27 | 0.01 |
| does not own a cell phone | 17% | 1% | 4% | 10% | -1.64 | 0.02 | -1.82 | 0.04 |
| **Bicycle** |  |  |  |  |  |  |  |  |
| owns a Bicycle | 9% | 4% | 8% | 8% | 1.65 | 1.65 | 1.65 | 1.65 |
| does not own a Bicycle | 91% | 96% | 92% | 92% | -0.13 | -0.13 | -0.13 | -0.13 |
| **Toilet type** |  |  |  |  |  |  |  |  |
| Flush to sewage | 45% | 70% | 53% | 29% | 0.68 | 0.02 | 2.26 | 0.04 |
| Flush to septic tank | 2% | 16% | 1% | 24% | 0.28 | 0.00 | 1.72 | 0.02 |
| Chemical | 1% | 3% | 2% | 2% | -2.99 | 0.01 | -0.58 | 0.00 |
| VIP | 12% | 3% | 11% | 15% | -1.79 | 0.01 | -0.29 | 0.00 |
| Pit without ventilation | 27% | 5% | 31% | 24% | -2.65 | 0.03 | -0.07 | 0.00 |
| Bucket | 5% | 1% | 0% | 3% | -3.21 | 0.02 | -1.13 | 0.00 |
| None | 9% | 1% | 1% | 3% | -4.04 | 0.03 | -2.59 | 0.03 |
| Other | 0% | 0% | 1% | 0% | -4.11 | 0.00 | -0.44 | 0.00 |
| **Main Walls Material** |  |  |  |  |  |  |  |  |
| Mud | 5% | 1% | 3% | 3% | -3.65 | 0.01 | -2.60 | 0.04 |
| Mud/cement | 6% | 20% | 6% | 6% | -3.26 | 0.01 | -2.32 | 0.05 |
| Corrugated iron/zinc | 15% | 18% | 10% | 10% | -2.74 | 0.10 | -1.13 | 0.01 |
| Prefab/wood | 6% | 1% | 1% | 1% | -1.68 | 0.01 | -1.25 | 0.00 |
| Bare brick/cement blocks | 25% | 22% | 78% | 78% | 0.71 | 0.03 | 0.76 | 0.04 |
| Plaster/finished | 42% | 37% | 1% | 1% | 0.61 | 0.00 | -1.48 | 0.00 |
| Other | 1% | 0% | 1% | 1% | -1.56 | 0.00 | -1.74 | 0.00 |
| **Dwelling Type** |  |  |  |  |  |  |  |  |
| House/concrete block | 51% | 33% | 61% | 72% | 0.77 | 0.03 | 0.73 | 0.03 |
| Traditional | 5% | 0% | 15% | 11% | -1.46 | 0.00 | -2.14 | 0.08 |
| Flat | 17% | 3% | 1% | 2% | 0.41 | 0.00 | -0.25 | 0.00 |
| Cluster house | 1% | 5% | 0% | 1% | 0.82 | 0.00 | 0.23 | 0.00 |
| backyard dwelling | 6% | 31% | 2% | 4% | 0.07 | 0.00 | 0.15 | 0.00 |
| Informal | 10% | 12% | 14% | 4% | -2.21 | 0.03 | -1.72 | 0.01 |
| Informal squatter | 10% | 10% | 6% | 6% | -3.39 | 0.09 | -1.66 | 0.01 |
| Room on property | 0% | 5% | 2% | 1% | -0.44 | 0.00 | 0.24 | 0.00 |
| Caravan/tent | 0% | 1% | 0% | 0% | -0.49 | 0.00 | -2.33 | 0.00 |
| Other | 0% | 0% | 0% | 0% | -1.40 | 0.00 | 0.14 | 0.00 |
| **Source of water** |  |  |  |  |  |  |  |  |
| Piped inside dwelling | 36% | 30% | 28% | 41% | 0.91 | 0.04 | 1.80 | 0.05 |
| Piped inside yard | 31% | 55% | 44% | 31% | -0.58 | 0.01 | 0.55 | 0.01 |
| Piped community stand | 18% | 14% | 21% | 16% | -3.70 | 0.09 | -0.64 | 0.01 |
| No access to piped water | 1% | 1% | 2% | 3% | -3.78 | 0.01 | -0.87 | 0.00 |
| Borehole | 1% | 0% | 1% | 2% | -3.97 | 0.00 | 0.29 | 0.00 |
| Open source | 7% | 0% | 3% | 6% | -2.50 | 0.00 | -1.94 | 0.04 |
| Other | 5% | 0% | 1% | 1% | -4.03 | 0.01 | -0.67 | 0.00 |

####

##### Supplementary Table 10 Regression coefficients for household income prediction

|  | **Quantile Regression**  **(25^th^ quantile; Log)** | **GLM regression (gamma log)** |
| --- | --- | --- |
| Constant | 4.26*** (0.06) | 5.24*** (0.08) |
| Urban | 0.15*** (0.04) | -0.01* (0.04) |
| Female | 0.07* (0.03) | 0.04* (0.03) |
| Educated ≥ grade 8 | 0.27*** (0.04) | 0.31*** (0.04) |
| Married / cohabitating | 0.21*** (0.04) | 0.20*** (0.04) |
| Has TB | -0.28*** (0.04) | -0.27** (0.10) |
| Employed | 0.33*** (0.03) | 0.33*** (0.04) |
| Asset quintile (ref Q1) |  |  |
| Quintile 2 | 0.20*** (0.04) | 0.25*** (0.03) |
| Quintile 3 | 0.48*** (0.05) | 0.57*** (0.04) |
| Quintile 4 | 0.73*** (0.04) | 0.73*** (0.04) |
| Quintile 5 | 1.37*** (0.05) | 1.66*** (0.06) |
| Age group (ref age 15-29) | |  |
| 30-44 | -0.09** (0.04) | -0.19*** (0.03) |
| 45 and over | 0.10* (0.05) | 0.10* (0.05) |
| Province (ref: Eastern Cape) | |  |
| Free State | 0.04* (0.07) | -0.19* (0.13) |
| Gauteng | 0.26*** (0.05) | -0.09* (0.13) |
| Mpumalanga | 0.13* (0.06) | 0.13* (0.11) |
| Western Cape | 0.26*** (0.05) | -0.08* (0.14) |
| KwaZulu-Natal | 0.24*** (0.04) | 0.10* (0.10) |
|  |  |  |
| N | 16,396 | 16,396 |
| Pseudo R2 | 0.18 |  |
| AIC |  | 24947.96 |
| Shapiro-Wilk test  for normality of residuals | 1.00*** | 0.97*** |

*** p < 0.001; ** p < 0.01; * p < 0.05

##### Supplementary Figure 6 Variation of regression coefficients across quantiles

##### Supplementary Table 11 Correlation coefficients for predicted and self-reported income

|  | **Quantile Regression** | **GLM Regression** |
| --- | --- | --- |
| Self-reported individual income: symptom onset  (collected in MERGE dataset) | 0.42*** | 0.33*** |
| Self-reported individual income: diagnosis  (collected in MERGE dataset) | 0.39*** | 0.29*** |
| Self-reported individual income: intensive phase  (collected in XTEND dataset) | 0.24** | 0.25*** |
| Self-reported individual income: continuation phase  (collected in XTEND dataset) | 0.21** | 0.23** |
| Self-reported household expenditure  (collected in REACH dataset) | 0.33*** | 0.34*** |

*** p < 0.001; ** p < 0.01; * p < 0.05

[1] Filmer D, Pritchett L. Estimating Wealth Effects Without Expenditure Data--Or Tears: An Application to Educational Enrollments in States of India. Demography 2001;38:115–32.

[2] Leibbrandt M, Woolard I, De Villiers L. Methodology: Report on NIDS Wave 1. Cape Town: 2009.

[3] Vyas S, Kumaranayake L. Constructing socio-economic status indices: how to use principal components analysis. Health Policy Plan 2006;21:459–68.

[4] Howe LD, Hargreaves JR, Huttly SRA. Issues in the construction of wealth indices for the measurement of socio-economic position in low-income countries. Emerg Themes Epidemiol 2008;5:1–14.

[5] Booysen F, van der Berg S, Burger R, Maltitz M von, Rand G du. Using an Asset Index to Assess Trends in Poverty in Seven Sub-Saharan African Countries. World Dev 2008;36:1113–30.

[6] Rutstein S. The DHS: Approaches for Rural and Urban Areas 2008.

[7] Kilian R, Matschinger H, Loeffler W, Roick C, Angermeyer MC. A comparison of methods to handle skew distributed cost variables in the analysis of the resource consumption in schizophrenia treatment. J Ment Heal Policy Econ 2002;5:21–31.

[8] Ataguba JE, Akazili J, McIntyre D. Socioeconomic-related health inequality in South Africa: evidence from General Household Surveys. Int J Equity Health 2011;10:48.

[9] Lonnroth K, Jaramillo E, Williams BG, Dye C, Raviglione M, Lönnroth K, et al. Drivers of tuberculosis epidemics: the role of risk factors and social determinants. Soc Sci Med 2009;68:2240–6.

[10] de Villiers L, Brown M, Woolard I, Daniels RC, Leibbrandt M. National Income Dynamics Study Wave 3 User Manual. *Cape Town: Southern Africa Labour and Development Research Unit.* 2013.

[11] Duan N. Smearing estimate: a nonparametric retransformation method. J Am Stat Assoc 1983;78:605–10.
